# Comprehensive host-pathogen protein-protein interaction network analysis

**DOI:** 10.1186/s12859-020-03706-z

**Published:** 2020-09-10

**Authors:** Babak Khorsand, Abdorreza Savadi, Mahmoud Naghibzadeh

**Affiliations:** 1 Computer Engineering Department, Faculty of Engineering, Ferdowsi University of Mashhad, Mashhad, Iran; 2 Ferdowsi University of Mashhad, Azadi Square, Mashhad, 9177948974 Iran

**Keywords:** Pathogen-host protein interaction network, Network analysis, Bipartite network, Centrality, Essential proteins

## Abstract

**Background:**

Infectious diseases are a cruel assassin with millions of victims around the world each year. Understanding infectious mechanism of viruses is indispensable for their inhibition. One of the best ways of unveiling this mechanism is to investigate the host-pathogen protein-protein interaction network. In this paper we try to disclose many properties of this network. We focus on human as host and integrate experimentally 32,859 interaction between human proteins and virus proteins from several databases. We investigate different properties of human proteins targeted by virus proteins and find that most of them have a considerable high centrality scores in human intra protein-protein interaction network. Investigating human proteins network properties which are targeted by different virus proteins can help us to design multipurpose drugs.

**Results:**

As host-pathogen protein-protein interaction network is a bipartite network and centrality measures for this type of networks are scarce, we proposed seven new centrality measures for analyzing bipartite networks. Applying them to different virus strains reveals unrandomness of attack strategies of virus proteins which could help us in drug design hence elevating the quality of life. They could also be used in detecting host essential proteins. Essential proteins are those whose functions are critical for survival of its host. One of the proposed centralities named diversity of predators, outperforms the other existing centralities in terms of detecting essential proteins and could be used as an optimal essential proteins’ marker.

**Conclusions:**

Different centralities were applied to analyze human protein-protein interaction network and to detect characteristics of human proteins targeted by virus proteins. Moreover, seven new centralities were proposed to analyze host-pathogen protein-protein interaction network and to detect pathogens’ favorite host protein victims. Comparing different centralities in detecting essential proteins reveals that diversity of predator (one of the proposed centralities) is the best essential protein marker.

## Background

Killing millions of humans, infectious diseases are the most brutal enemies of the entire history. Billions of dollars are spent to reveal the way hosts are infected by pathogens and their presumptive victims. Host-Pathogen protein-protein interactions can be the best clue for initiating infection which have been studied in different pathogens [1–8]. Exploring molecular functions, biological processes, cellular compartment, common pathways and the other properties of host proteins targeted by pathogens can help us in infectious disease inhibition. Investigating common targets of different pathogens could help us to design multipurpose drugs.

Moreover, investigating protein-protein interactions between human proteins (HPPIs) could help us to find viruses’ potential HPs victims. To do that, HPPI network is analyzed by different centrality measures. In network analysis, centrality is the main concept of identifying gravity of each node in the network. Centrality measures can be used to find most important HPs in HPPI network (HPPIN) to identify new drug targets [9–15]. Each centrality measure defines nodes’ weight from a different perspective. HPPIN has been analyzed by different centralities such as Degree Centrality [16], Closeness [17], Lobby Index [18], Betweenness [19], Clustering Coefficient [20], Leader Rank [21], Topological Coefficient [22], Module Centrality [23], Eigenvector Centrality [24], Neighborhood Connectivity [25], Normalized Alpha Centrality [26], Average Shortest Path Length [27], Subgraph Centrality [28], Radiality [29], Range limited Centrality [30] and Eccentricity [31].

Essential genes are minimal gene sets which are indispensable for a living cell and their functions are the foundation of life [32]. As disruption of these genes can lead to cell death, we investigate human virus protein-protein interaction network (HVPPIN) to see whether the product of essential genes (essential proteins) are main targets of virus proteins (VPS) and which human proteins (HPs) are targeted by more VPs.

In this paper, we focus on human as host and integrate experimentally protein-protein interactions (PPIs) between human proteins and virus proteins (HVPPIs) from different databases. Exploring the HVPPIN shows that human proteins which are targeted by different virus strains either have a lot of interactors in intra HPPIN or bridge between two large cliques.

Additionally, network analysis is performed on HPPIN by eight different centralities. Results demonstrate that centrality scores of HPs targeted by different virus strains are significantly higher than the other HPs. Besides, it reveals that centrality scores of essential proteins (EPs) are significantly higher than non-essential proteins.

HVPPIN is a special type of network called bipartite in which interactions are interspecies in contrast with HPPIN which is a unipartite network meaning interactions are intraspecies. As most of the common centralities are designed for unipartite network and HVPPIN is a bipartite network, seven novel bipartite centralities including CHTV (connectivity of human proteins targeted by same virus protein), PS (propagation speed), DP (diversity of predators), DSP (decreased shortest path), CI (component index), CR (crown centrality) and VC (vulnerable centrality) are proposed to analyze HVPPIN.

CHTV and CR scores of HVPPIN demonstrate the significant higher scores of real samples in comparison with random ones. PS analysis reveals the property of three degree of separation for HPPIN in presence of virus proteins. DSP results disclose the robustness of HPPIN. By doing extra analysis on VC, we found that virus proteins that have higher VC score, target considerably more EPs in comparison with virus proteins with lower VC score and could be chosen for drug target purposes. Finally, comparing DP scores of EPs and non-EPs with other centrality scores of EPs and non-EPs reveals that DP out performs the other centralities in detecting EPs and could be used as EPs marker.

Moreover, we make a database for human proteins centralities, DP score and virus families evolutionary distance which is publicly available at http://bioinf.modares.ac.ir/software/PHINA.

## Methods

### Preliminaries and definitions

A *graph* is generally illustrated by *G*(*V*, *E*) where *V*(*G*) is the set of nodes also known as vertex set and *E*(*G*) ⊂ *V*(*G*) ⨯ *V*(*G*) is the set of edges between the graph’s nodes also known as edge set. Two vertices *v*_*i*_ and *v*_*j*_ which are connected by an edge are called *adjacent*. All the vertices adjacent to *v*_*i*_ are called the neighbors of *v*_*i*_ and declared by *N*(*v*_*i*_). Number of neighbors of *v*_*i*_ is called *degree* of *v*_*i*_ and denoted by *deg*(*v*_*i*_) = |*N*(*v*_*i*_)∣. Edges which have the same end vertices are called parallel edges and if both end vertices are the same, it will be called a self-loop. A graph which has neither parallel edges nor a self-loop is called a simple graph, otherwise it is called multigraph or hyper graph. A sequence of alternating vertices is called *walk*. Walk with no repeated edge is called *trial* and trial with sets of unique vertices is called *path*. If a path exists between each pair of graph vertices, it is called *connected* graph. *Subgraph* of a graph is a graph *S*(*V*, *E*) which *V*(*S*) ⊆ *V*(*G*) and *E*(*S*) ⊆ *E*(*G*). Maximally connected subgraph of a graph is called *component*. A fully connected graph is said *complete* graph. A complete subgraph of a graph is called *clique* [33].

A *bipartite* graph [34] *G*(*T*, *B*, *E*) is a kind of graph that has two vertices disjointing subset *T* and *B* (top and bottom) which *V*(*G*) = *T*(*G*) ⋃ *B*(*G*) and *T*(*G*) ⋂ *B*(*G*) = ⌀. Moreover, each edge has an end vertex from the top node and another from bottom nodes (∀(*tb*) ∊ *E*(*G*) ∣ *t* ∊ *T*(*G*), *b* ∊*B*(*G*)). In a bipartite graph for any top vertex *t*_*i*_ ∊ *T*(*G*), *N*(*t*_*i*_) ⊂ *B*(*G*) while for any bottom vertex *b*_*i*_ ∊ *B*(*G*), *N*(*b*_*i*_) ⊂ *T*(*G*). There are many real bipartite networks such as actor-movie network [35] which illustrates the movies and the actors playing in them, author-article network [36] which shows the articles and theirs authors, gene-disease network [37–43] which reveals diseases and their targeted genes, metabolite-enzyme network [44–46] which shows metabolites and their corresponding enzymes, and host-pathogen PPI network such as fungal pathogen scedosporium aurantiacum with human long epithelial cells [47], *Leptospira interrogans* and *Homo sapiens* [48], extracellular bacterial pathogen and human host [49], zika virus non-structural proteins and human host proteins [50], bacterial and fungal pathogens and maize stalks host [51].

**Fig. 1:**
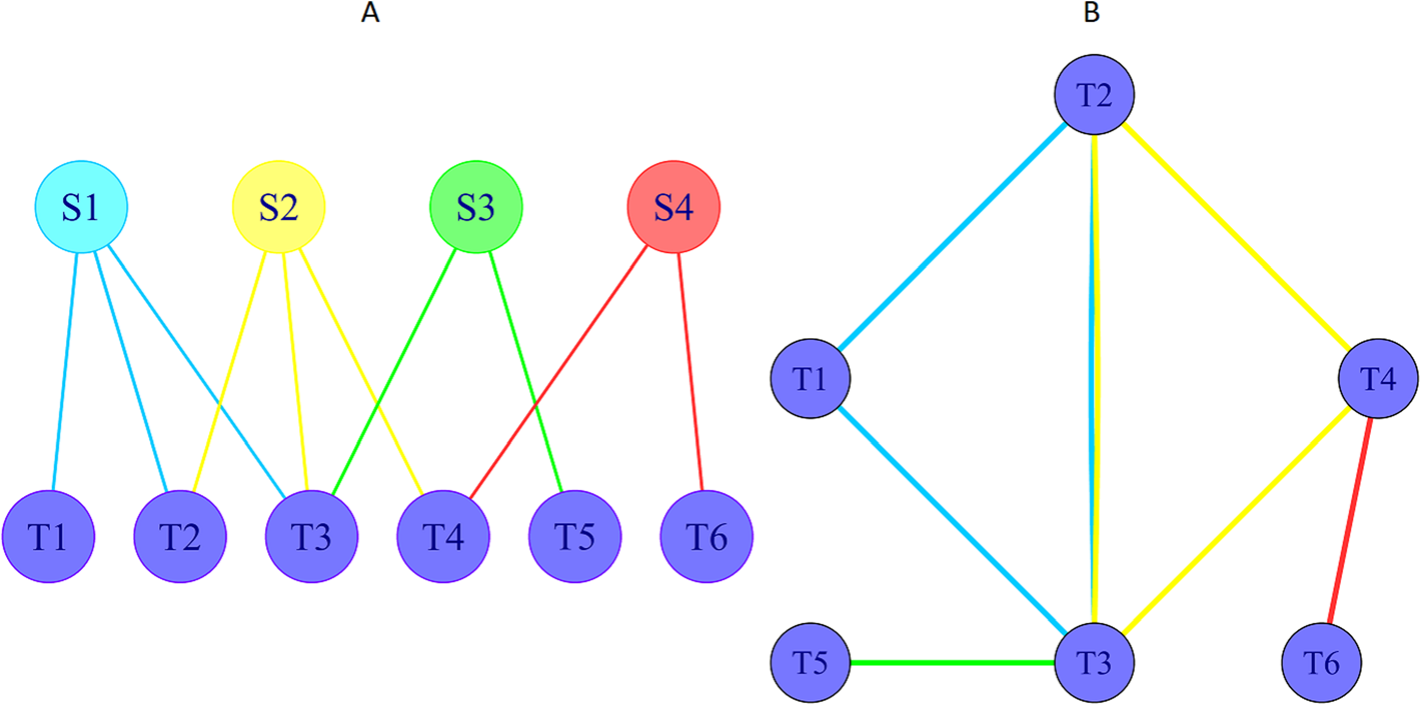
Bipartite graph and its projection. Left graph shows a bipartite graph with 4 top nodes and 6 bottom nodes. Right graph illustrates the projection of the bipartite graph. Each top node in bipartite graph makes a clique in projection graph. Top nodes of the bipartite graph are colored by different colors to distinguish the related cliques

Bipartite *projection* of graph *G*(*T*, *B*, *E*) is graph *G* ′ (*B*^′^, *E*′) where nodes are subsets of *G* bottom nodes which have at least one common neighbor. Each pair of nodes that have common neighbors are connected with an edge as it is shown in sample graph of Fig 1. In other words, each top node makes a clique with the size of its degree.

**Fig. 2:**
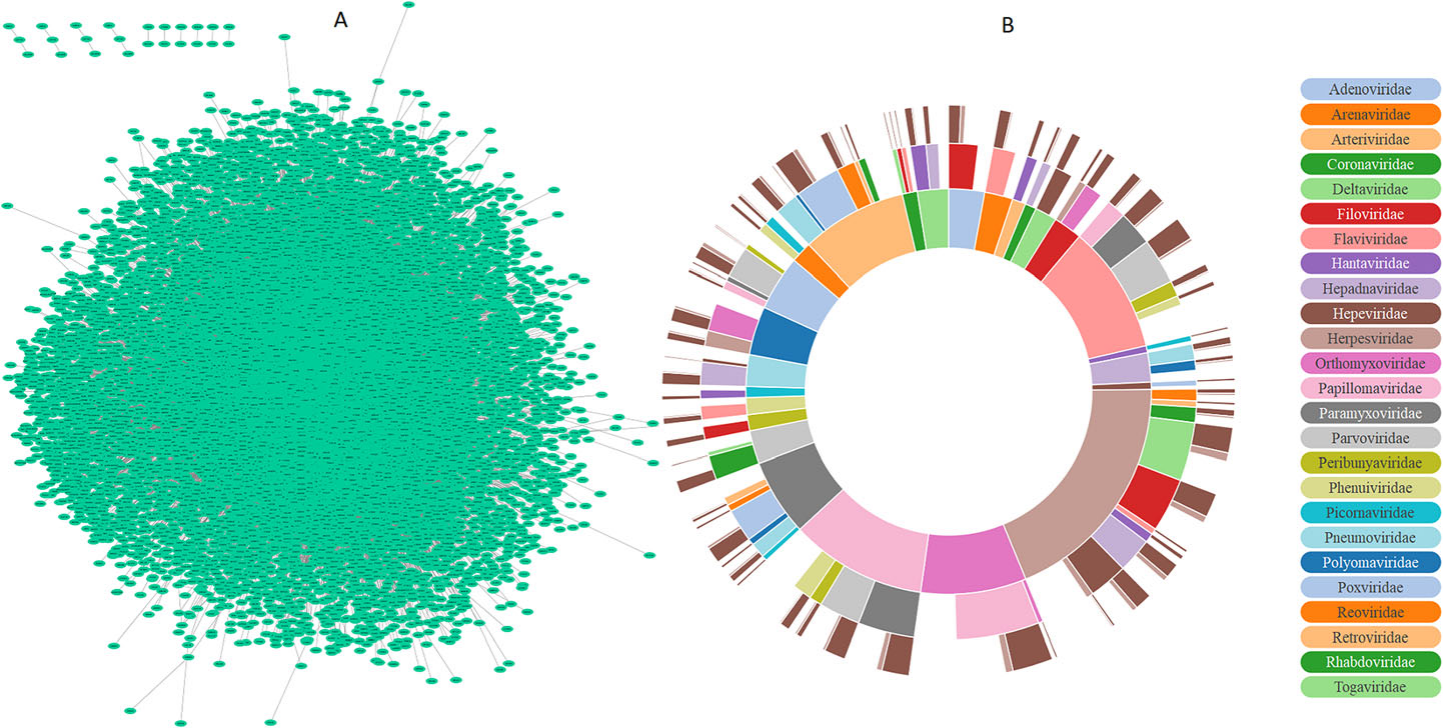
**a** Human protein-protein interaction network (HPPIN). 262,820 Interactions between 19,995 human proteins. 24 HPs in 10 components and remaining 19,971 HPs in one main component. **b** Sunburst chart of HVPPIN human and virus proteins distribution. The distribution of virus families and genus are respectively shown in the first and second layer. The number of human and virus proteins are shown in the last layer by dark and light brown colors respectively

Human protein-protein interaction network (HPPIN) is a graph, the nodes of which are human proteins (HPs) and interaction between them are illustrated by graph edges. There are many public repositories for molecular interaction data [52]. We extracted 262,814 HPPIs from Intact [53] and BioGrid [54] including 19,995 HPs. Most of biological networks have one main component. As it is shown in Fig. 2-A, more than 0.999 of HPs are in one main component which reveals the existence of a path between 99.9% HP pairs in HPPIN.

Human-virus protein-protein interaction network (HVPPIN) is a bipartite graph in which virus and human proteins are its top and bottom nodes respectively and interaction between them are illustrated by graph edges. We extracted 34,768 HVPPIs from Intact [53], VirusMint [55], DIP [56], STRING [57], and BioGrid [54] including 7141 HPs and 1281 VPs which belong to 34 different families, 88 different types of genus and 236 different strains as depicted in Fig. 2-B.

In the proposed article, we investigated the following famous centralities in HPPIN:

**DC (Degree Centrality):** For each HP, DC is the number of its interacting partners. Virus proteins can infect many of HPs by targeting an HP with a high degree.

**NC (Neighborhood Connectivity):** For each HP, NC is the average degree of all its neighbors. Virus proteins can infect many of HPs by targeting an HP with the high NC through its neighbors.

**SP (Average Shortest Path Length):** The length of a path is the number of interactions which will be traversed. The pass with minimum length between each two HPs is considered as the shortest path. For each HP as shown in the following formula, SP is the summation of the shortest path between that HP and all the other HPs divided by the number of HPs. ${SP}_p=\left({\sum}_{m=1}^n{S}_{p,m}\right)/n$. HPs with low SP can be considered as good targets for virus proteins. By reaching those HPs, virus proteins can quickly propagate to the other HPs.

**TC (Topological Coefficients):** The extent to which an HP shares neighbors with others. Zero would be assigned to HPs that have less than two neighbors. TC is defined as: ***TC***_***p***_ = ***avg***(***J***_***p***, ***m***_)/***k***_***p***_, where *J*_*p*. *m*_ is defined for all HPs *m* that share at least one neighbor with HP *p* and the value *J*_*p*. *m*_ is the number of neighbors shared between the HPs *p* and *m*, plus one if there is a direct link between them. However, *k*_*p*_ is the degree of *p*.

**CS (Closeness Centrality):** The reciprocal of the average shortest path length. It is, in fact, a number between 0 and 1 which is computed as: ***CS***_***p***_ = **1**/***avg***(***L***_***p***, ***m***_).

where *L*_*p*. *m*_ is the length of the shortest path between two HPs *p* and *m*.

Zero would be assigned to isolated HPs. This measure shows Propagation speed of viruses from a given HP to the other reachable ones in the HPPIN.

**CC (Clustering Coefficients):** For each HP, CC is the number of triangles passing through it, relative to the maximum number of triangles that could pass through. ***CC***_***p***_ = **2*e***_***p***_/***k***_***p***_(***k***_***p***_ − **1**)

where *k*_*p*_ is the degree of *p* and *e*_*p*_ is the number of connected pairs between all its neighbors.

**BC (Betweenness Centrality):** The amount of control which one HP exerts over the interactions of others in the HPPIN and it is defined as follows: ***BC***_***p***_ =  ∑ ***σ***_***st***_(***p***)/***σ***_***st***_, where *s* and *t* are HPs in the HPPIN different from *p*, *σ*_*st*_ shows the number of shortest paths from *s* to *t*, and *σ*_*st*_ (*p*) is the number of shortest paths from *s* to *t* that *p* lies on.

**RD (Radiality):** For each HP, RD is calculated by subtracting SP from the HPPIN diameter. Hence, HPs with higher RD are usually closer to the other nodes, whereas, HPs with lower RD are peripheral.

**EV (Eigenvector centrality):** EV is calculated by the eigenvector of the largest eigenvalue of adjacency matrix. It is a measure to declare the influence level of an HP node within HPPIN. HPs with high EV have a wide-reaching influence on HPPIN.

**PR (Page rank centrality):** For each HP, it measures the importance of HPs connected to that HP. It is equal to the sum of page rank score of its neighbors.

As it is shown in Fig. 4-C, for all of the 19,995 HPs of HPPIN, all of the mentioned centralities are calculated and reported in our database which is publicly accessible at the following address: http://bioinf.modares.ac.ir/software/PHINA/Centrality.php

Moreover, we propose seven new centralities for analyzing HVPPIN:

### Connectivity of the human nodes targeted by the same virus node (CHTV)

For each virus node, human nodes targeted by that node (HTV) are chosen. CHTV is the number of connected pairs of HTVs in HPPIN over all possible pairs and reached by the following formula:

$$ {CHTV}_i={\sum}_{j=1}^n{\sum}_{k=j+1}^n{A}_{jk} $$

*CHTV*_*i*_ is connectivity of the human proteins targeted by *ith* virus protein, *n* is the number of *HTV*_*i*_*s* and *A*_*jk*_ is a binary function with one value in the cases where an edge exists between *H*_*j*_ and *H*_*k*_ and zero otherwise.

**Fig. 3:**
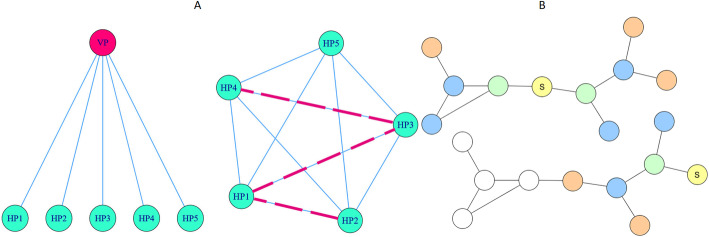
**a** For each virus protein, a clique was made by all of its HPs’ targets. Connectivity of the human nodes targeted by the same virus node (CHTV) is the number of interactions between the elements of the clique in human protein-protein interaction network (HPPIN) (pink lines) divided by number of clique edges (blue lines). In this example, CHTV will be 0.3. **b** Propagation Speed (PS) illustrates the percentage of infected human proteins (HPs) in three levels. At each level, PS is the Sum of the number of infected HPs up to that level and their neighbors divided by the number of all HPs. By assuming yellow node labeled by S as the first infected human protein, green nodes are the first group of human proteins which will be infected by S, thus PS1 score is 0.3 and 0.2 for the first and second networks, respectively. The blue nodes will be infected by the green nodes while the orange nodes will be infected by blue nodes

As it is shown in Fig. 3-A, all the targeted nodes by the same source node will construct a clique in projection network. Hence, CHTV will show the fraction of clique edges which contribute in real world network of targeted nodes.

### Propagation speed

Propagation speed (PS) is a measure which illustrates the speed of propagation of a virus in HPPIN or a rumor in society. We defined three levels of PS. Each level indicates the percentage of infected human proteins in that round. So PS1 is the number of human proteins targeted by virus protein and their neighbors over the number of all human proteins in HPPIN. PS2 is the number of infected human proteins of level one plus all of their neighbors over the number of all human proteins in HPPIN. Finally, PS3 is the number of infected human proteins of level two plus all of their neighbors over the number of all human proteins in HPPIN.

As an example, consider node S as the first infected node in two different depicted networks of Fig. 3-B. PS1, PS2 and PS3 for the first network (network at the top of the figure) is 0.3, 0.7 and 1, respectively. PS1, PS2 and PS3 for the second network (network at the bottom of the figure) is 0.2, 0.4 and 0.6, respectively. Score 1 for PS3 of the first network reveals that all of the network nodes are infected while score 0.6 shows that 60% of the second network nodes are infected.

In HPPIN, HTVs are chosen. Cumulative percentage of the number of HTVs’ first neighbors, second neighbors and third neighbors are PS1, PS2 and PS3 scores, respectively. In other words, PS1, PS2 and PS3 indicate the percentage of human proteins of HPPIN which will be infected within the first, second and third round of interactions, respectively.

Among all 34,768 interactions in HVPPIN, 28639 interactions containing virus strains with at least 5 interactions are chosen. The final HVPPIN contains 1093 virus proteins belonging to 26 different families, 67 different types of genus and 124 different strains. For each strain separately, HTVs are chosen and PS1, PS2 and PS3 scores are calculated.

### Diversity of predators

In HVPPIN, HPs, which are targets of VPs of different families, are chosen. For each of these HPs, each of the virus families is called a predator. Evolutionary distance (ED) among any couple of predators of each HP is calculated. For each HP, mean of EDs of its predators multiplied by the number of its predators is considered as its diversity of predators (DP) score. As an example, Q9Y6H1 is targeted by VPs of four different virus families (Matonaviridae (M), Flaviviridae (F), Herpesviridae (H) and Papillomaviridae (P)). To calculate its DP, we need to calculate $$ \left(\begin{array}{c}n\\ {}2\end{array}\right) $$ EDs between each couple of its predators where n is the number of predators. Therefore, for the mentioned example, we need to calculate $$ \left(\begin{array}{c}4\\ {}2\end{array}\right)=6 $$ Eds and the DP will be calculated as follows:

$$ \left({ED}_{M,F}+{ED}_{M,H}+{ED}_{M,P}+{ED}_{F,H}+{ED}_{F,P}+{ED}_{H,P}\right)/6\ast 4 $$

**Fig. 4:**
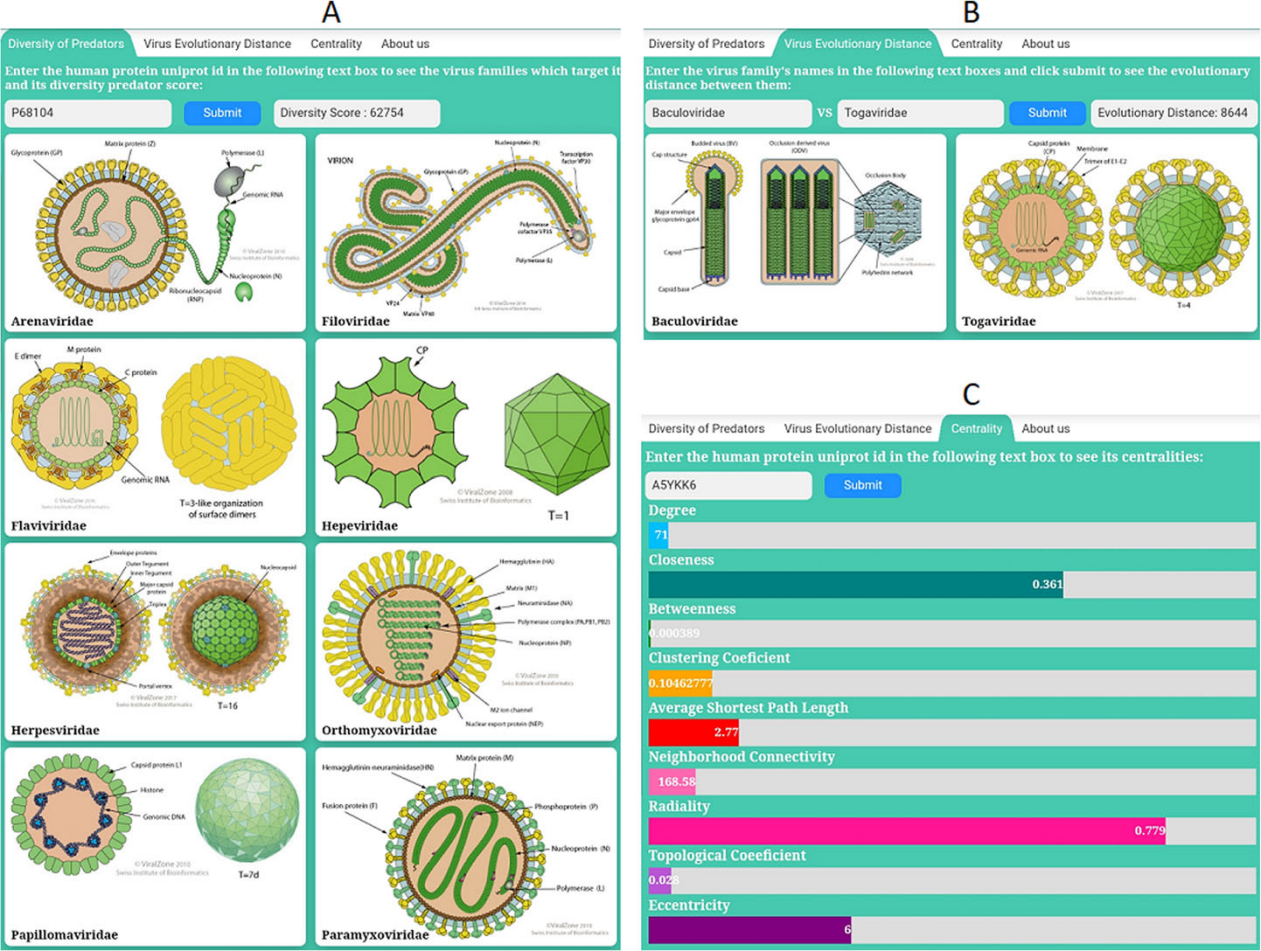
**a** Diversity of Predators: By entering the uniprot id of a human protein (HP) and pressing the submit button, all virus families which have an interactor with that HP will be depicted (from ViralZone, SIB Swiss Institute of Bioinformatics licensed under a creative common attribution 4.0 international license) and the diversity score will be reported. **b** Virus Evolutionary Distance: By entering two virus families in the text boxes and pressing submit button, evolutionary distance between them will be reported. **c** By entering uniprot id of an HP and pressing submit button, centralities of that HP in HPPIN will be reported. In each centrality, the number shows the real centrality score while the progress bar shows its normalized score in [0,1]

As there is not any database for ED among virus families, we create a database for calculating ED of the most famous virus families. To calculate ED between two virus families, we make Needleman-Wunsch [58] global pairwise alignment between each VP of the first family with all VPs of the second one. Mean of alignment scores of all pairs is considered as ED between the two families. As an example, for calculating ED between Orthomyxoviridae with 132 different VPs and Papillomaviridae with 106 VPs, 13,992 (132*106) global pairwise alignments were calculated and mean of all these alignments were reported as ED of these two families. Our database (Fig. 4-B) is publicly accessible at the following address: http://bioinf.modares.ac.ir/software/PHINA/EvolutionaryDistance.php

Among all 6500 HPs targeted by different VPs in HVPPIN, 3141 HPs interact with VPs of at least two virus families. For all of these HPs, DP is calculated and reported in our database (Fig. 4-A) which is publicly accessible at the following address: http://bioinf.modares.ac.ir/software/PHINA/VirusFamilies.php

(All the pictures of virus families inside our site are gathered from *ViralZone:**www.expasy.org/**viralzone, SIB Swiss Institute of Bioinformatics* [59] *which is licensed under a creative common attribution 4.0 international*.)

### Decreased shortest path

In the main HPPIN component the shortest path between each two HP pair is calculated. Afterwards, HVPPIs of one of the virus strains is added to HPPIN and the shortest path between each two HP pairs is recalculated. Difference between sum of all possible HP pairs’ shortest path in presence or absence of HVPPIs is measured as decreased shortest path (DSP).

Among all 32,859 interactions in HVPPIN, 28306 interactions containing virus proteins with at least 2 interactions are chosen. The final HVPPIN contains 784 virus proteins belonging to 25 different families, 64 different types of genus and 111 different strains. For each of these strains, DSP is calculated and DSPs’ mean of each family’s strain is considered as DSP of that family. The same was performed for random cases in a way that random nodes with the same degree distribution of real strains were added to HPPIN and DSP were calculated.

### Component index

Component index is the tendency of HPs targeted by VPs of one genus to interact with each other rather than the HPs targeted by VPs of another genus. First, induced sub graph (ISG) of HPs targeted by all VPs of each strain was extracted from HPPIN. Then, for each strain, the number of interactions within its ISG HPs, and the number of interactions between its HPs and other ISG HPs was calculated. Finally, component index was defined between two strains by the following formula:

$$ \left({\sum}_{i=1}^{n_{ic}}{\sum}_{j=i}^{n_{ic}}{e}_{ij}-{\sum}_{i=1}^{n_{ic}}{\sum}_{o=1}^{n_{oc}}{e}_{io}\right)/\left({\sum}_{i=1}^{n_{ic}}{\sum}_{j=i}^{n_{ic}}{e}_{ij}+{\sum}_{i=1}^{n_{ic}}{\sum}_{o=1}^{n_{oc}}{e}_{io}\right) $$

*n*_*ic*_ is the number of first ISG HPs, *n*_*oc*_ is the number of second ISG HPs, *e*_*ij*_ is 1 if *ith* HP of first ISG interacts with *jth* HP of the first ISG. *e*_*io*_ is 1 if *ith* HP of first ISG interacts with *oth* HP of second ISG. In other words, component index is the difference between the intra-interactions of an ISG and inter-interactions of that ISG with another ISG over the sum of inter and intra interactions.

### Crown centrality

**Fig. 5:**
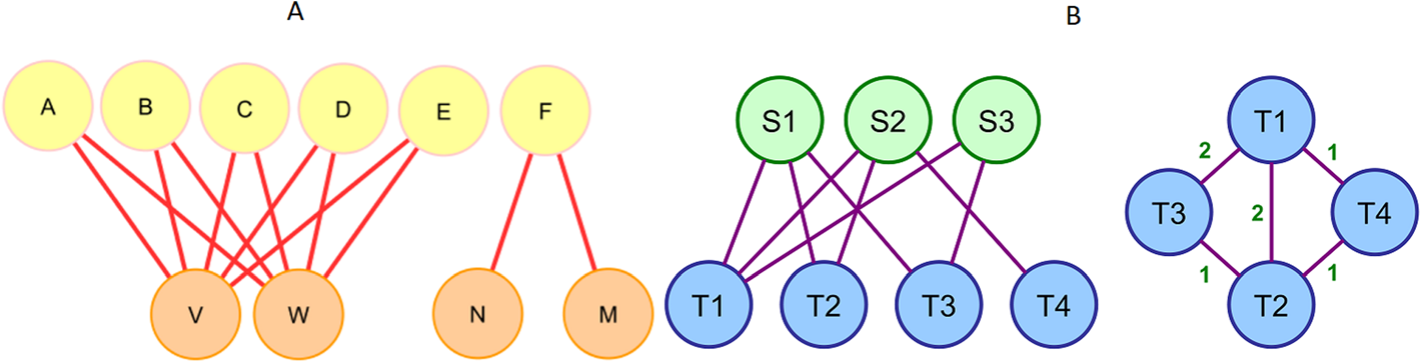
**a** Crown centrality. Virus proteins (yellow nodes) which target the same pairs of HPs (orange nodes) make a crown. VW is a crown with five ivories while NM is a crown with one ivory. **b** Vulnerable Centrality. It is defined for each top node as division of sum of reciprocal of related clique edges weight in projection by its size

Virus proteins which target the same pair of HPs formed a crown. Number of virus proteins which target the same pair of HPs determine the number of ivories of a crown (Fig. 5-A). Number of ivories of a crown equals the degree of node reached by vertex contraction of HP pairs. Existence of crowns in HVPPIN shows the tendency of virus proteins to be cooperative in attacking HPs. Crowns with higher number of ivories shows the more important interaction between HP pairs of that crown. HP pairs of a crown which are adjacent in HPPIN has the clustering coefficient 1.

Among all 34,768 interactions in HVPPIN, 27859 interactions containing virus proteins with a degree of more than one are chosen. The final HVPPIN contains 990 virus proteins belonging to 19 different families, 42 different types of genus and 67 different strains.

We investigated the final HVPPIN for the crowns and did the same for random models with the same degree distribution.

### Vulnerable centrality

As projection of a bipartite network is a simple graph and there are many different available centralities to analyze it, one way of analyzing bipartite network is analyzing its projection.

The main problem of this idea is the information loss in converting a bipartite network to its projection.

We define vulnerable centrality to reflect the effect of each top node in bottom-projection. As an example, as it is shown in Fig. 5-B, edge *T*_1_*T*_4_ is just made by *S*_2_ while *T*_1_*T*_3_ is made by both *S*_1_ and *S*_3_ in projection. Thus, by elimination of *S*_3_, edge *T*_1_*T*_3_ remains, though by elimination of *S*_2_, edge *T*_1_*T*_4_ will be permanently dropped from projection.

Vulnerable centrality is defined for each top node as sum of reciprocal of projection weight divided by the equivalent clique size. As an example, for the graph of Fig. 5-B, *V*_1_. *V*_2_. *V*_3_ (vulnerable score for *S*_1_. *S*_2_. *S*_3_) is calculated by:

$$ {V}_1=\left(\frac{1}{1}+\frac{1}{2}+\frac{1}{2}\right)/3=0.67.{V}_2=\left(\frac{1}{1}+\frac{1}{1}+\frac{1}{2}\right)/3=0.83.{V}_3=\left(\frac{1}{2}\right)/1=0.5 $$

Essential proteins functions are indispensable for life cycle and thus, investigating them can be useful for both drug target purposes for inhibition and better understanding of their behaviors. 2472 EPs were gathered from Georgi [60], 1734 EPs were reported in Blomen [61], 1878 EPs were extracted from Wang [62], 3230 EPs were derived from Lek [63] and 7127 were collected from Chen [64]. Finally, 6115 genes which were at least reported in two of the mentioned studies were considered as essential proteins.

Lots of papers apply network topology for detecting new essential proteins [65–70]. Eight different mentioned centralities and two new proposed centralities (DP and VC) were calculated for all of the HPs of HPPIN. Afterwards, centralities of essential HPs were compared against non-essential HPs and the results were reported to investigate whether they could be applied for detecting new essential proteins.

Furthermore, Virus proteins of each strain, were clustered into 2 groups based on vulnerable score. Thereafter, the number of essential proteins targeted by each group were compared to see whether there exists a significant difference between the two groups.

## Results

All the existing mentioned centralities and the two new proposed centralities (DP and VC) are investigated to see whether they could be an essential protein marker.

For the other five proposed centralities, different topics were investigated. For CHTV and crown centrality, we figured out if there is a significant difference in real sample scores in comparison with random sample scores or not. We also uncovered the degree of separation of HPPIN. Moreover, robustness of HPPIN is measured by DSP. Propensity of HPs targeted by the same VP to have inter or intra interaction was investigated.

### Essential proteins are critical nodes in HPPIN

**Fig. 6:**
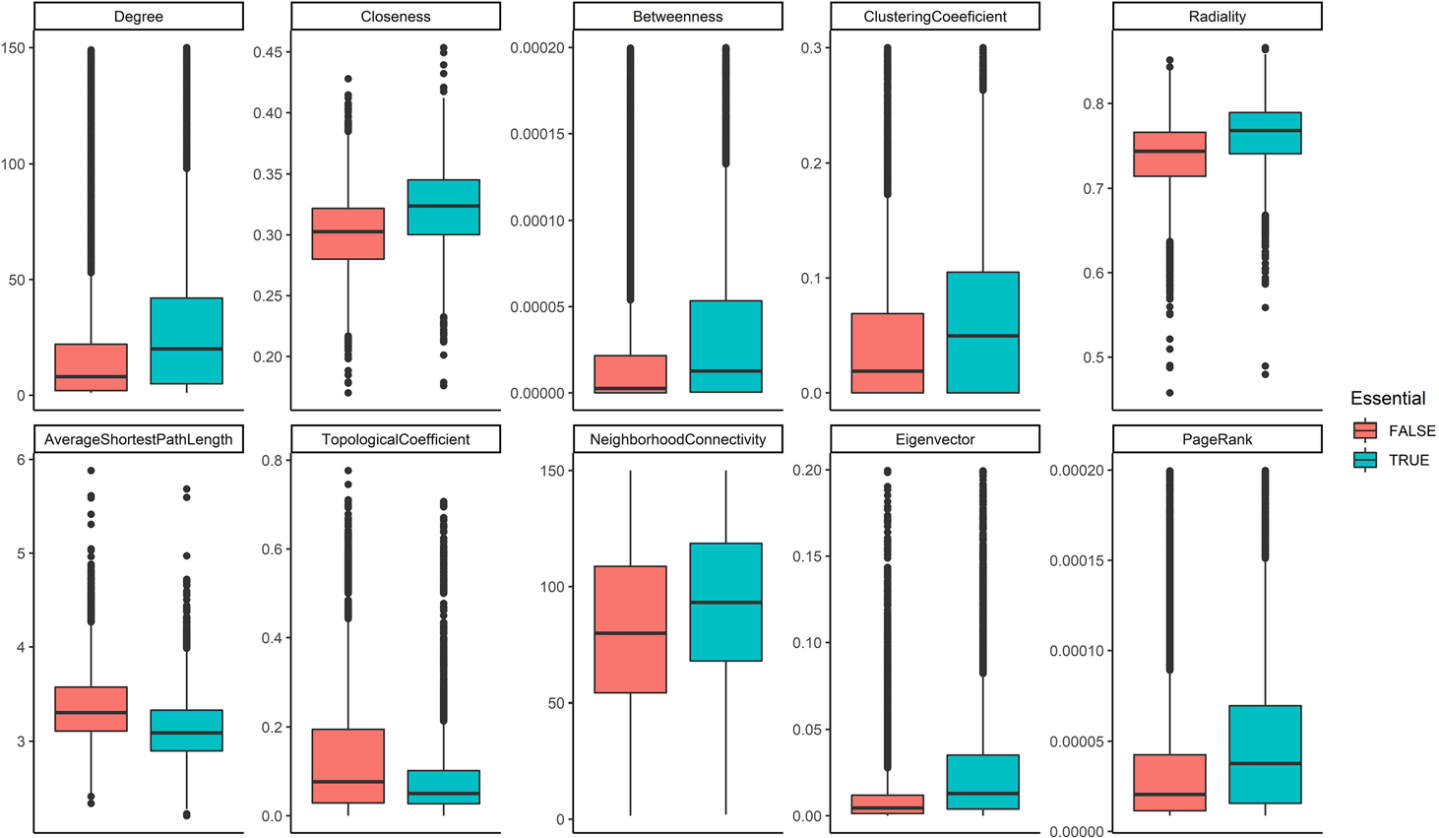
Comparison of centrality scores of essential proteins versus non-essential proteins

Ten different centralities were measured in HPPIN and as it is shown in Table 1 and Fig. 6, essential proteins’ centrality scores were significantly different in comparison with non-essential proteins.

**Table 1:** Comparison of centrality scores of essential proteins versus non-essential proteins

Centrality	Essential Proteins		Non-Essential Proteins		t-score	*p*-value
	Mean	Standard Error	Mean	Standard Error		
Degree	38.41	0.84	20.12	0.33	20.14	2.7e-88
Radiality	0.76	0.0004	0.73	0.0003	38.68	9.4e-311
Closeness	0.32	0.0004	0.3	0.0002	40.7	1.2e-313
Betweenness	0.0002	0.00002	0.00007	0.000003	6.45	1.2e-10
Clustering Coefficient	0.095	0.0017	0.082	0.0014	5.78	7.9e-09
Average Shortest Path	3.15	0.004	3.36	0.003	−38.68	9.4e-311
Topological Coefficient	0.096	0.0015	0.138	0.0013	−20.55	1.1e-92
Neighborhood Connectivity	233.93	4.84	206.64	3.28	4.66	3.1e-06
Eigen vector centrality	0.03	0.0006	0.01	0.0002	29.02	1.8e-176
Page rank centrality	0.0007	0.00002	0.0004	0.000005	15.06	1.3e-50

### Virus proteins with higher VC scores have a huge tendency to target essential proteins

**Fig. 7:**
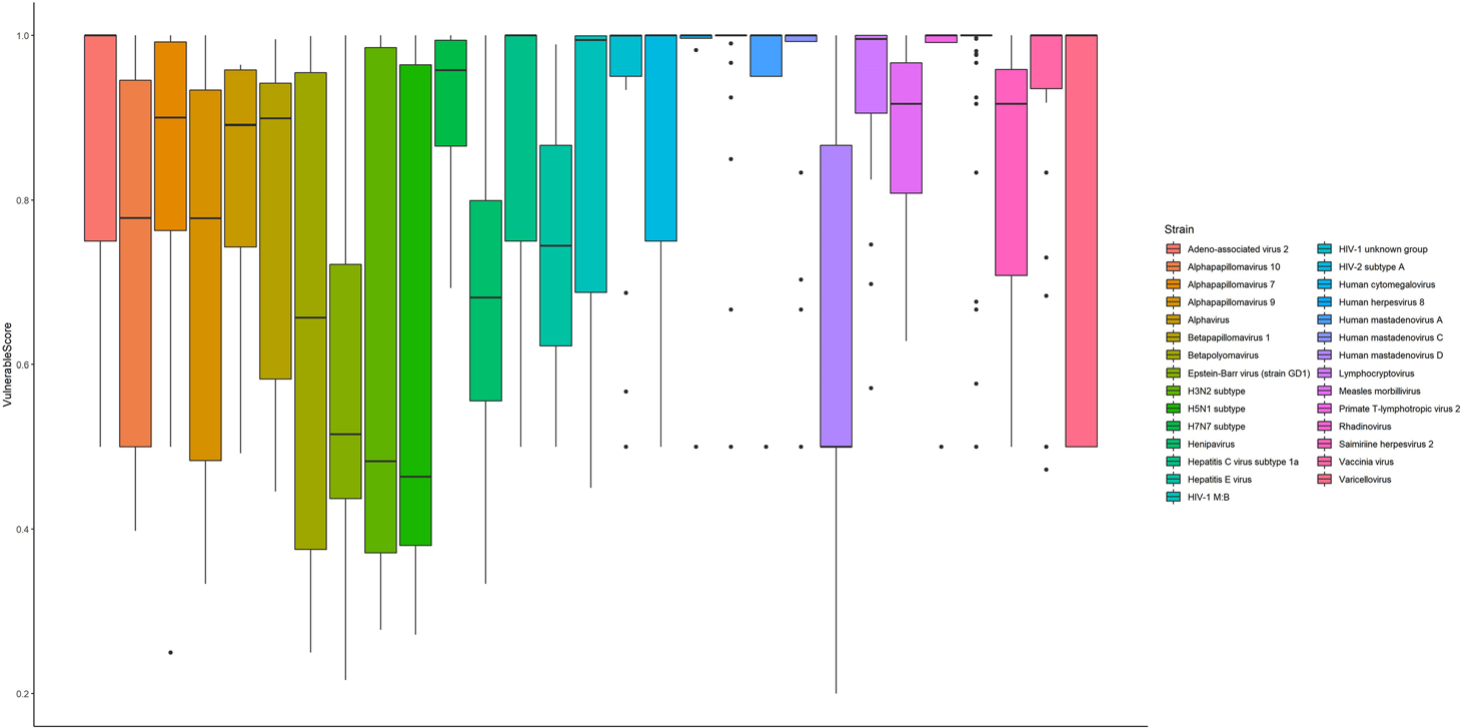
Vulnerable scores box plot of 29 different strains. Vulnerable centrality illustrates the effect of each virus protein in constructing the projection of infected human proteins. Thus, virus proteins with higher vulnerable score must be inhibited sooner than the others

In our model, vulnerable centrality illustrates the effect of each virus protein in constructing the projection of infected human proteins. Thus, virus proteins with higher vulnerable scores must be inhibited sooner than the others. In other words, inhibiting the virus proteins with higher vulnerable scores will maximized the chance of early inhibition of whole virus spread. As it is illustrated in Fig. 7, by calculating vulnerable scores of 29 different strains, virus proteins of each strain have a considerable diverse vulnerable score which makes this centrality capable of detecting the important virus proteins of each strain.

Moreover, virus proteins of each strain were separated into two groups according to VC scores. Essential HPs targeted by each group are reported in Table 2.

**Table 2:** Comparison of percentage of essential HPs targeted by virus proteins with high against low vulnerable scores

Strains	Total number of targeted HPs	Number of essential targeted HPs	Percentage of essential targeted HPs with low VC score	Percentage of essential targeted HPs with high VC score
Adeno-associated dependoparvovirus	255	191	0.01	1
Human betaherpesvirus 5	76	48	0.1	0.94
Ebolavirus	291	194	0.37	0.66
Betapapillomavirus 1	618	324	0.23	0.83
Dengue virus	164	104	0.04	0.96
H5N1	342	237	0.22	0.92
H7N7	61	42	0.4	0.95
Varicellovirus	10	6	0.33	0.83
Orf virus	4	4	0.75	1
Human mastadenovirus D	14	6	0.67	0.67
HEV	10	8	0.12	1
Equine infectious anemia virus	4	3	0.33	0.67
Human polyomavirus 2	44	27	0.07	0.96
Hendra henipavirus	62	18	0.11	0.94
Primate T-lymphotropic virus 2	61	24	0.04	1

As clearly manifested by the results, virus proteins with higher VC scores have a huge tendency to target essential proteins. Consequently, VPs with higher VC scores seem to be better drug targets in comparison with others VPs.

### DP centrality outperformed the other centralities in detecting essential proteins and potential drug targets

Among all 6500 HPs targeted by different VPs in HVPPIN, 3141 HPs interact with VPs of 34 different virus families. Table 3 shows 15 HPs with the highest DP score.

**Table 3:** Human proteins with the highest DP score. Columns three to six shows the number of distinct virus families, genus, species and proteins which target that human proteins, respectively

Human	Gene	Family	Genus	Species	Virus	DP
P09651	HNRNPA1	12	14	18	32	65,080
P68104	EEF1A1	15	20	27	49	62,754
P07437	TUBB	14	22	29	49	61,135
P07910	HNRNPC	12	12	14	27	60,101
P06748	NPM1	14	17	21	34	56,997
Q08211	DHX9	10	13	15	27	56,766
P22626	HNRNPA2B1	10	11	13	29	56,218
P08670	VIM	8	10	15	26	56,058
Q12805	EFEMP1	8	8	9	13	55,709
P62753	RPS6	10	11	12	23	55,552
P17844	DDX5	10	13	16	28	55,121
P05141	SLC25A5	12	14	18	28	54,483
P11940	PABPC1	9	11	16	30	54,245
P38159	RBMX	9	9	11	31	54,235
P61978	HNRNPK	10	13	15	33	54,026

Further analysis reveals that EPs’ DP scores are significantly higher than non-EPs’ DP score and it could be used as a marker for detecting EPs.

To prove our claim, we compare the ability of detecting EPs between the proposed centrality and all the other mentioned centralities. To do so, for each centrality, we picked 200 HPs with the highest score and counted the number of EPs to declare the sensitivity of that centrality. As it is shown in Table 4, DP centrality outperformed others in detecting EPs.

**Table 4:** Number of essential proteins detected by each centrality and its consequent sensitivity

	DP	EV	CS	RD	BC	DC	PR	NC	TC	CC	SP
Number of EPs out of 200	166	156	142	142	118	116	116	80	56	50	38
Sensitivity	0.83	0.78	0.71	0.71	0.59	0.58	0.58	0.4	0.28	0.25	0.19

To check the ability of different centralities in detecting potential drug targets, we chose two different gene expression data (GSE1739, GSE150316). Two hundred ninety nine and one hundred sixty two differentially expressed genes (DEG) were extracted from GSE150316 and GSE1739 respectively. Different centrality measures were calculated for them. In each centrality, the number of DEGs with centrality scores more than 3rd quantile score were reported in Table 5. This reveals the ability of each centrality in detecting potential drug targets.

**Table 5:** Number of potential drug targets detected by each centrality and its consequent sensitivity

	DP	EV	CS	RD	BC	DC	PR	NC	TC	CC	SP
Number of central HPs	212	193	196	196	170	175	168	103	31	94	75
Sensitivity (GSE150316)	0.71	0.64	0.65	0.65	0.56	0.58	0.56	0.35	0.1	0.32	0.26
Number of central HPs	131	125	125	125	107	115	110	68	27	53	66
Sensitivity (GSE1739)	0.81	0.77	0.77	0.77	0.66	0.71	0.68	0.42	0.16	0.33	0.41

### CHTV scores of real samples are considerably higher than random cases

By considering *n* as the number of different viruses, *n* CHTV scores have been separately calculated for each genus. Mean of CHTV score of each type has been reported as CHTV score of that type.

**Fig. 8:**
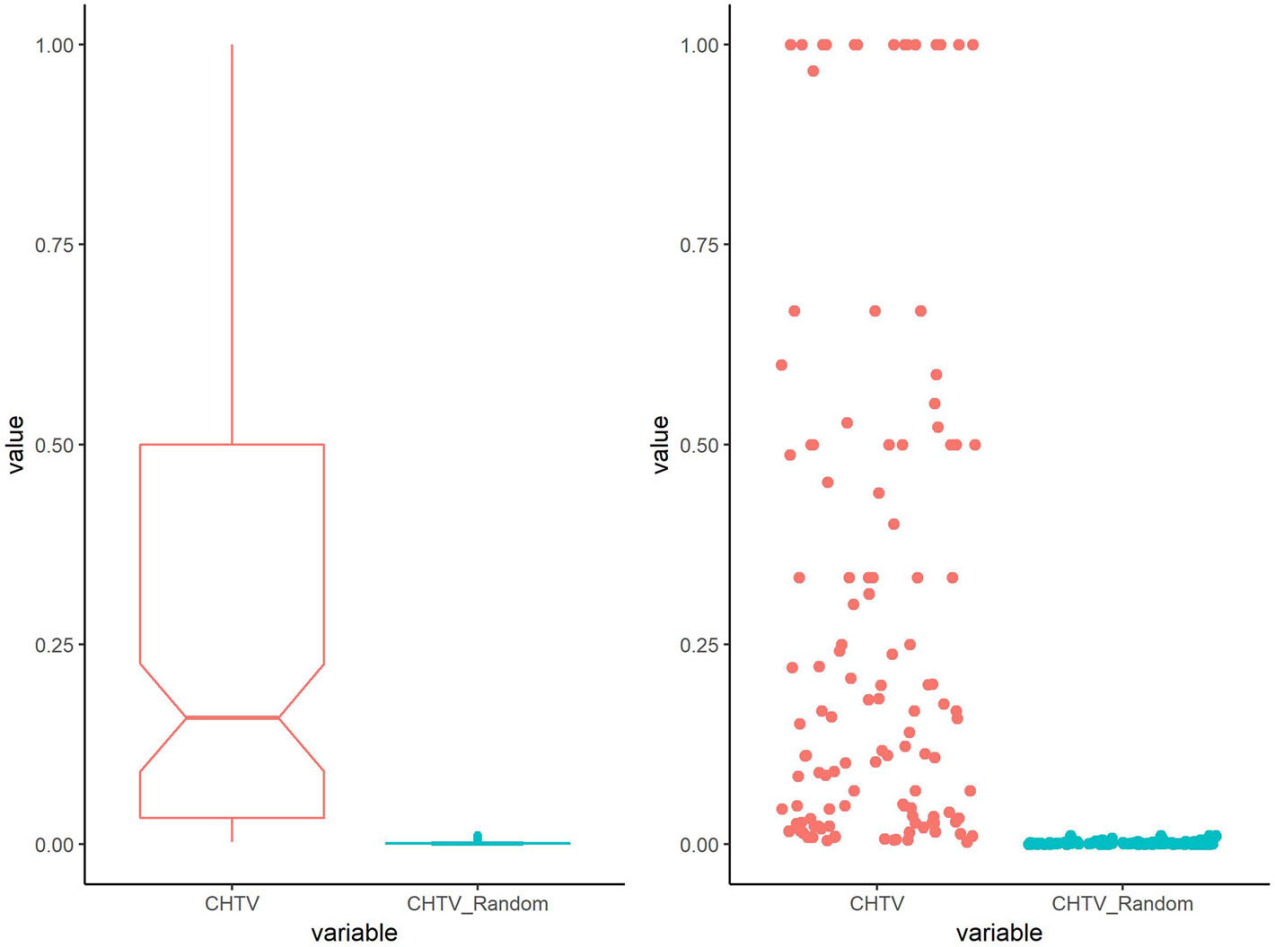
Box plot and scatter plot of CHTV scores of 64 different virus genera with red color and equivalent random classes with green color. As it is obvious in the plots, CHTV scores of real samples are considerably higher than random cases

Moreover, 64 Random classes were constructed equal to the number of genus types. For each class, *CHTV*_*i*_ shown in Fig. 8, is calculated for each virus protein of the related class by selecting random human proteins from human proteins of HPPIN equal to the number of *HTV*_*i*_*s*.

Furthermore, for comparing the results of CHTV scores of real and random samples, statistics score (z-score) is calculated for each class of virus genus by the following formula:

$$ {Z}_i=\frac{CHTV_i- mean\left( CHTV-{Random}_i\right)}{sd\left( CHTV-{Random}_i\right)} $$

where *Z*_*i*_ and *CHTV*_*i*_ indicate the z-score and CHTV score of *ith* genus, respectively and *CHTV* − *Random*_*i*_ shows the CHTV score of *ith* related genus.

**Fig. 9:**
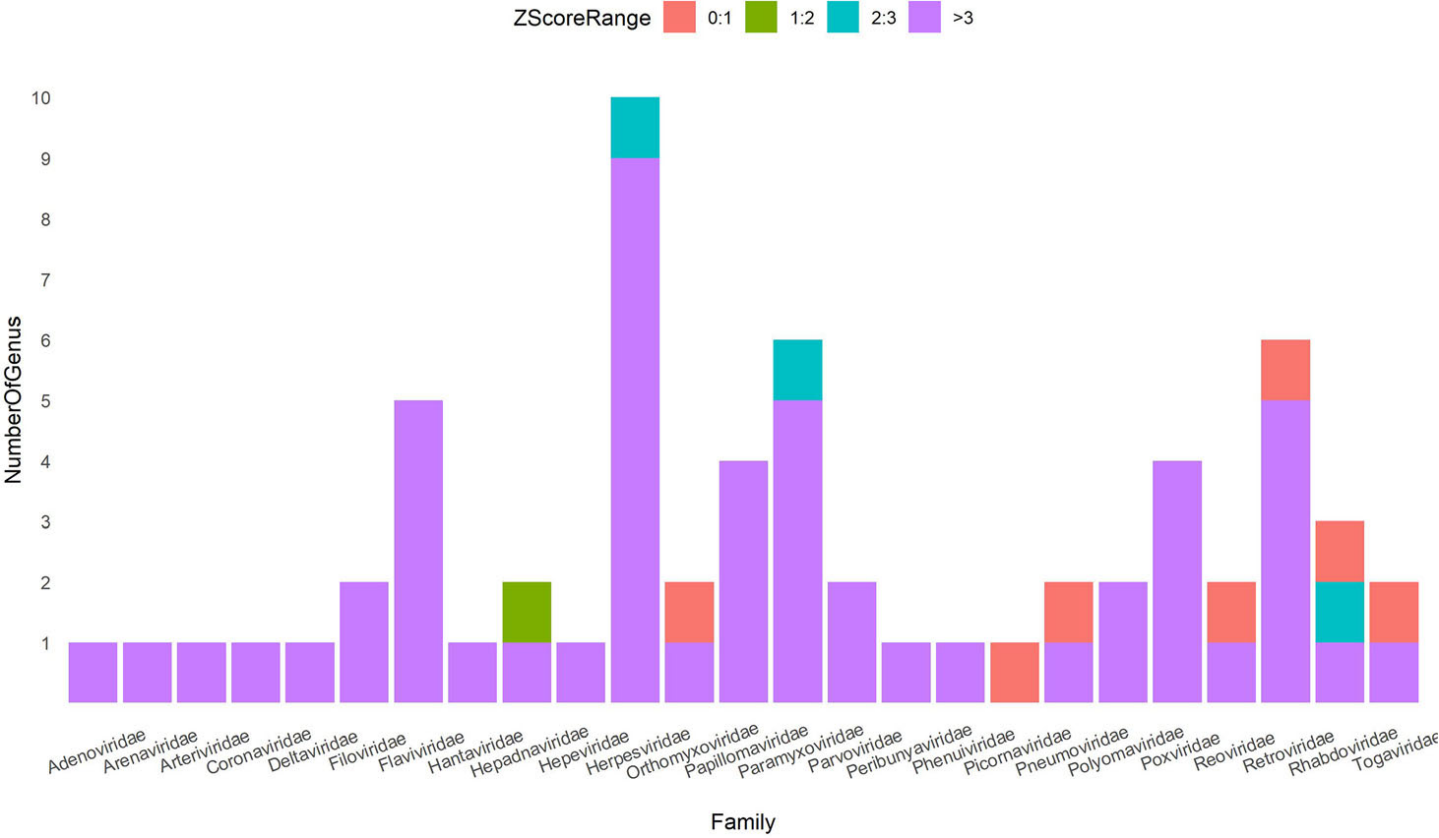
CHTV z-scores’ stack bar plot of different families. Colors illustrate the range of Z-scores of each genus. Teal and violet colors indicate Z-scores between 2 to 3 and above 3, respectively, showing statistical significance between real and random samples. Contrastively, red indicates there is not any difference and green shows that there is a little difference between random and real samples

The standard score *Z*_*i*_ shows the distance of CHTV score of *ith* genus from the mean of CHTV score of the related random class. As it is shown in Fig. 9, 83% of z-scores are more than 3 times of standard deviation above mean of random CHTV scores.

### Number of crowns with more than two ivories of real samples are substantive in comparison with random classes

**Fig. 10:**
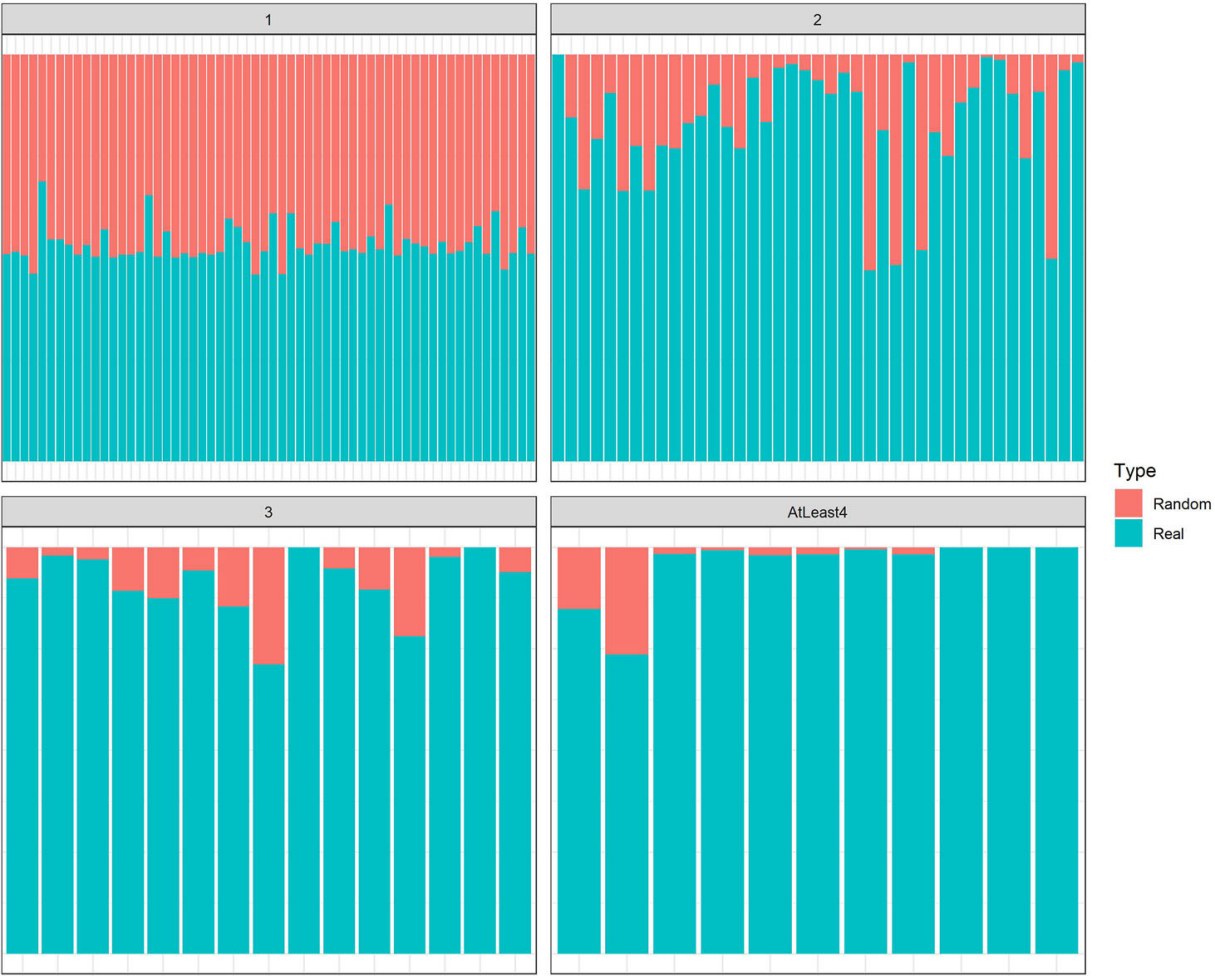
Stacked bar plot of crown centrality of 67 different strains and equivalent random classes. To compare crown frequency in real and random samples, number of crowns in each sample is divided by the sum of the frequency of both real and random samples. Top left and top right panels show the frequency of size one and two crowns respectively while bottom left and bottom right panels show the frequency of size three and more than three crowns, respectively. Crowns’ frequency of real and random samples are illustrated by red and teal color, respectively

Results are depicted in Fig. 9 for crowns with one, two, three and more than three ivories. As evident in Fig. 10, real model precedes random model in terms of percentage of crowns as well as the higher the number of ivories and the greater difference between real and random model.

### More than 70% of HPs are infected in just two rounds (two degree of separation)

**Fig. 11:**
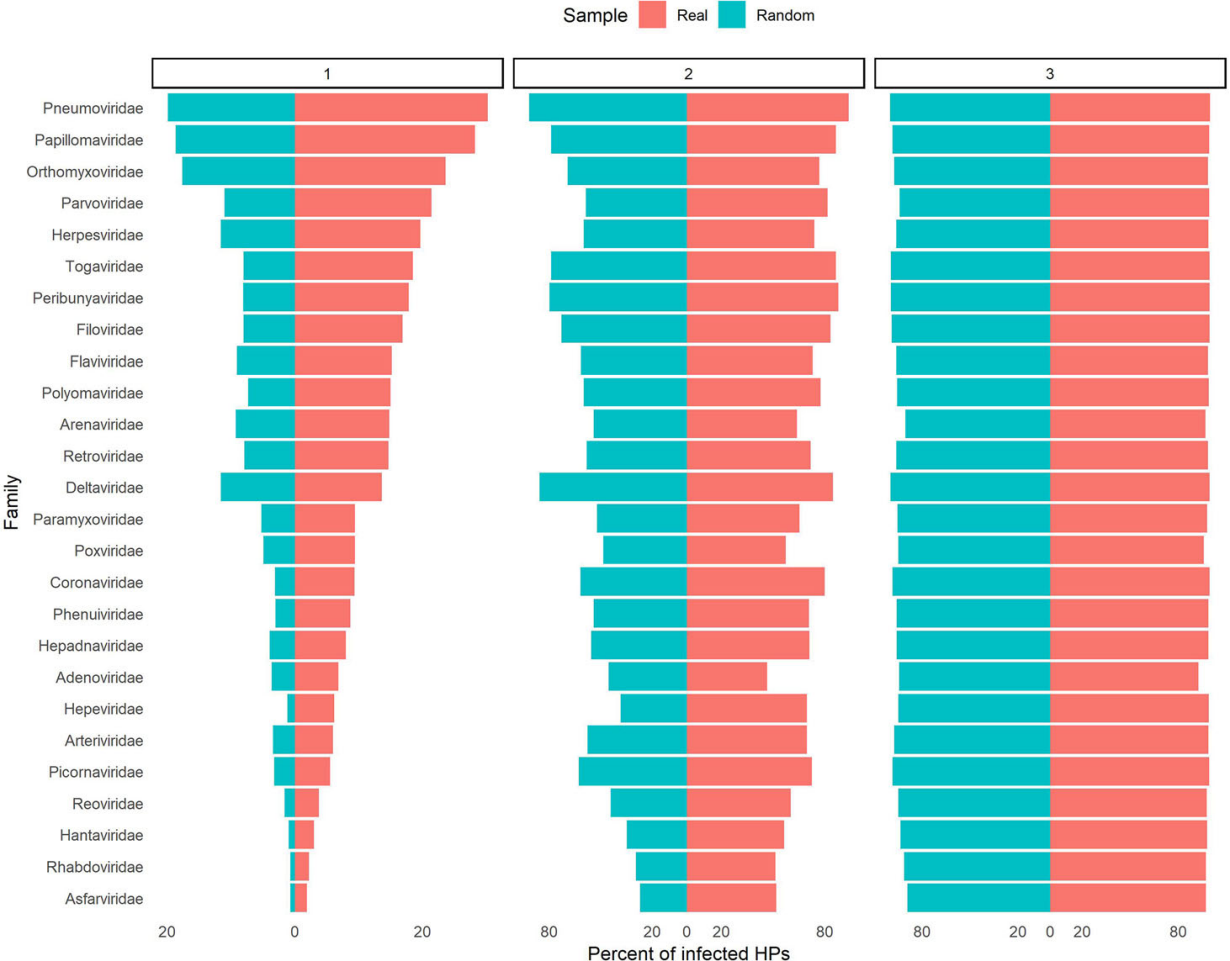
Bar plot of PS1, PS2 and PS3 scores of 26 different virus families and equivalent random classes. PS scores of real and random samples are illustrated by red and teal color, respectively

For each HVPPIN strain separately, HTVs are chosen and PS1, PS2 and PS3 scores are calculated. For each family, mean of each score is illustrated in Fig. 11 with red color.

Moreover, 26 classes containing 124 random classes were constructed equal to the number of families and strains. For each class, equal to the number of virus proteins’ targets of the related strain, random human proteins were selected as HTVs and PS1, PS2 and PS3 scores were calculated. For each family, mean of each score was illustrated in Fig. 10 with teal color.

**Fig. 12:**
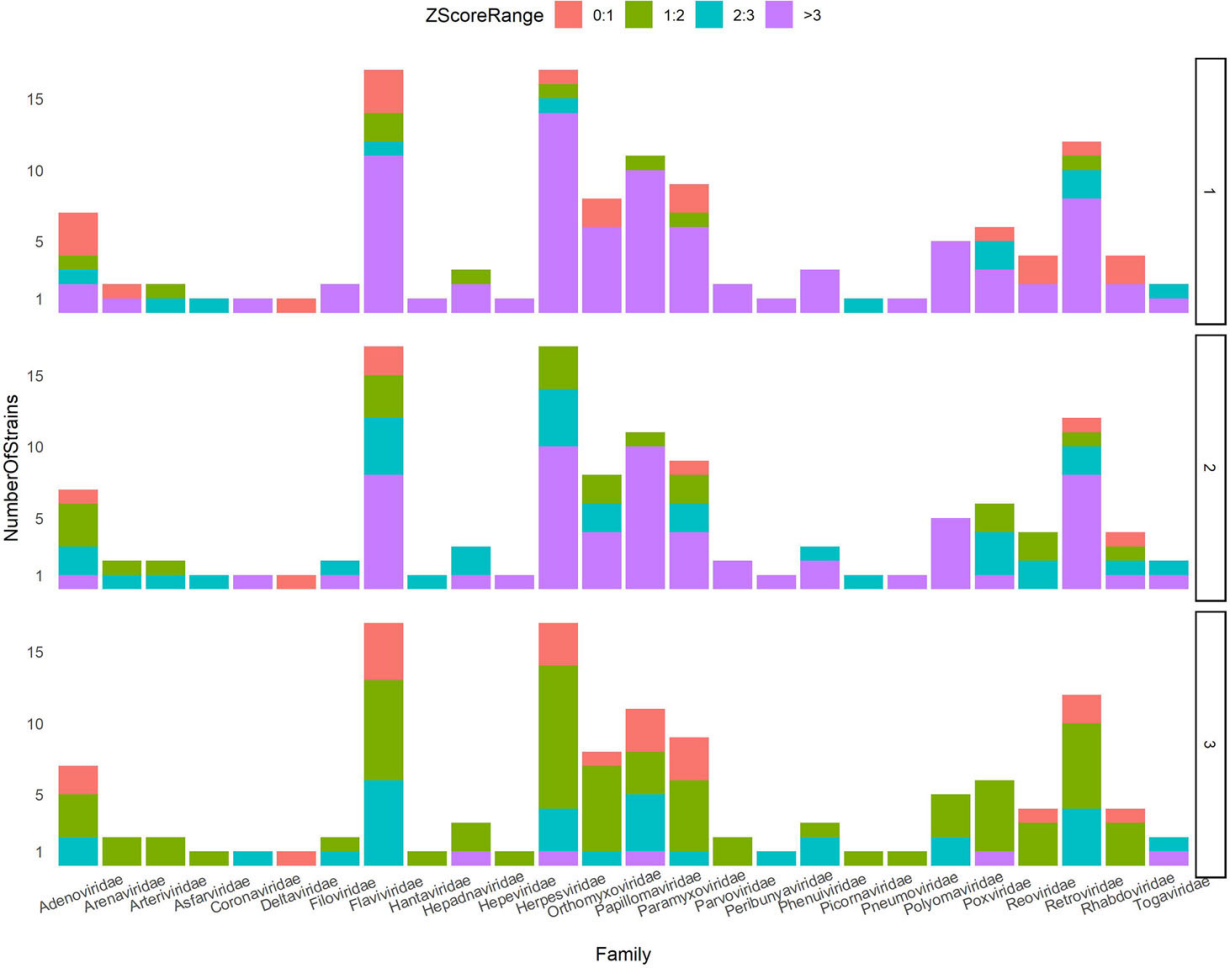
PS z-scores’ stack bar plot of different families. Panel 1, 2 and 3 show the z-scores extracted from PS1, PS2 and PS3 scores, respectively. Colors illustrate the range of z-scores of each genus. Teal and violet colors indicate z-scores between 2 to 3 and above 3, respectively which show statistical significance between real and random samples. Contrastively, red reveals there is not any difference and green shows that there is a little difference between random and real samples

Only in two rounds, in most of the virus families, more than 70% of HPs of HPPIN are infected. Furthermore, for comparing the results of PS scores of real and random samples, statistics score (z-score) was calculated for each class of virus as shown in Fig. 12.

### DSP illustrates the robustness of HPPIN

**Fig. 13:**
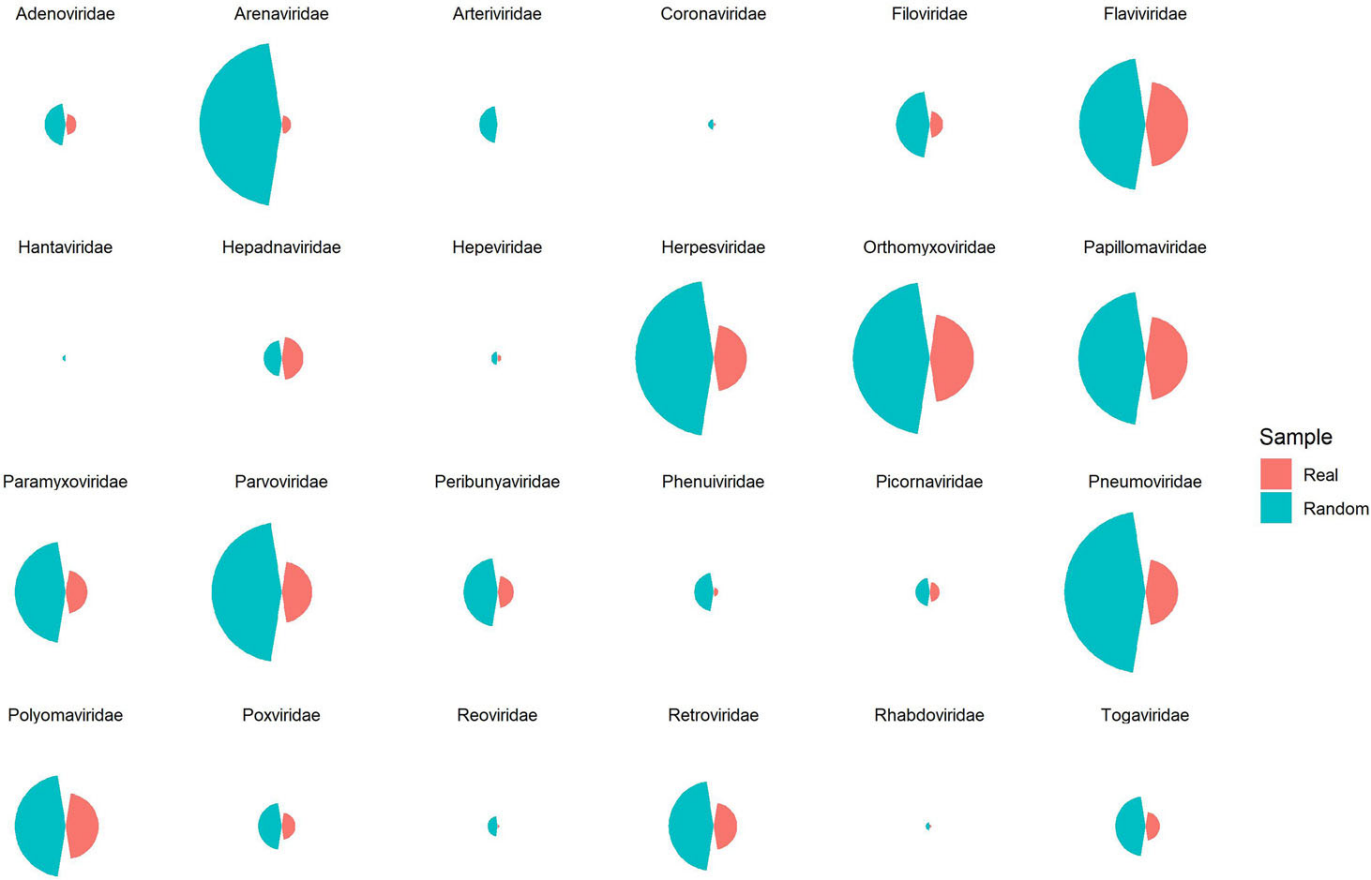
Mushroom plot of Decreased Shortest Path (DSP) of 25 different virus families and equivalent random classes. Number of shortest paths which decreased by appending virus proteins and their interactions with human proteins to HPPIN are measured with DSP. DSP scores of real and random samples are illustrated by red and teal colors, respectively

Watts-Strogats model [20] is a random model constructed by rewiring the edges of a regular ring lattice. Shortest path between pairs of a regular ring lattice is so larger than Watts-Strogats model because rewired edges act as shortcuts. As shown in Fig. 13, random classes thus far, have better DSP scores which is due to the shortcuts created by random choosing of HPs in random model, while in real models, as virus proteins target specific HPs in specific tissue, local shortest path reduction will happen. This result also shows the robustness of HPPIN.

### Most of strains have a positive component index

Component Index (CI) was calculated between all 1431 pairs of 54 strains which had at least 5 HVPPIs. Mean and median of all CIs were 0.62 and 0.71, respectively, which shows the great tendency of HPs targeted by each strain to interacts within its ISG and make most of them as a real component. As an example, in Table 6, Alphainfluenzavirus CI is calculated with the first 10 largest genera (in terms of the number of interactions). Alphainfluenzavirus has 2794 HPs and 27,296 interactions. The number of HPs and interactions of the other strains are placed in OCHPs and OCIs columns, respectively.

**Table 6:** Component Index of Alphainfluenzavirus against first 10 biggest genera

Genus 1	Genus 2	CI	OCIs	OCHPs
Alphainfluenzavirus	Lymphocryptovirus	0.589	7073	1082
Alphainfluenzavirus	Alphapapillomavirus	0.396	11,809	1634
Alphainfluenzavirus	Flavivirus	0.515	8745	1525
Alphainfluenzavirus	Lentivirus	0.652	5767	1020
Alphainfluenzavirus	Rhadinovirus	0.581	7240	812
Alphainfluenzavirus	Hepacivirus	0.586	7134	963
Alphainfluenzavirus	Betapapillomavirus	0.644	5911	750
Alphainfluenzavirus	Simplexvirus	0.748	3932	551
Alphainfluenzavirus	Morbillivirus	0.914	1221	465
Alphainfluenzavirus	Betapolyomavirus	0.766	3613	312

## Discussion

**Fig. 14:**
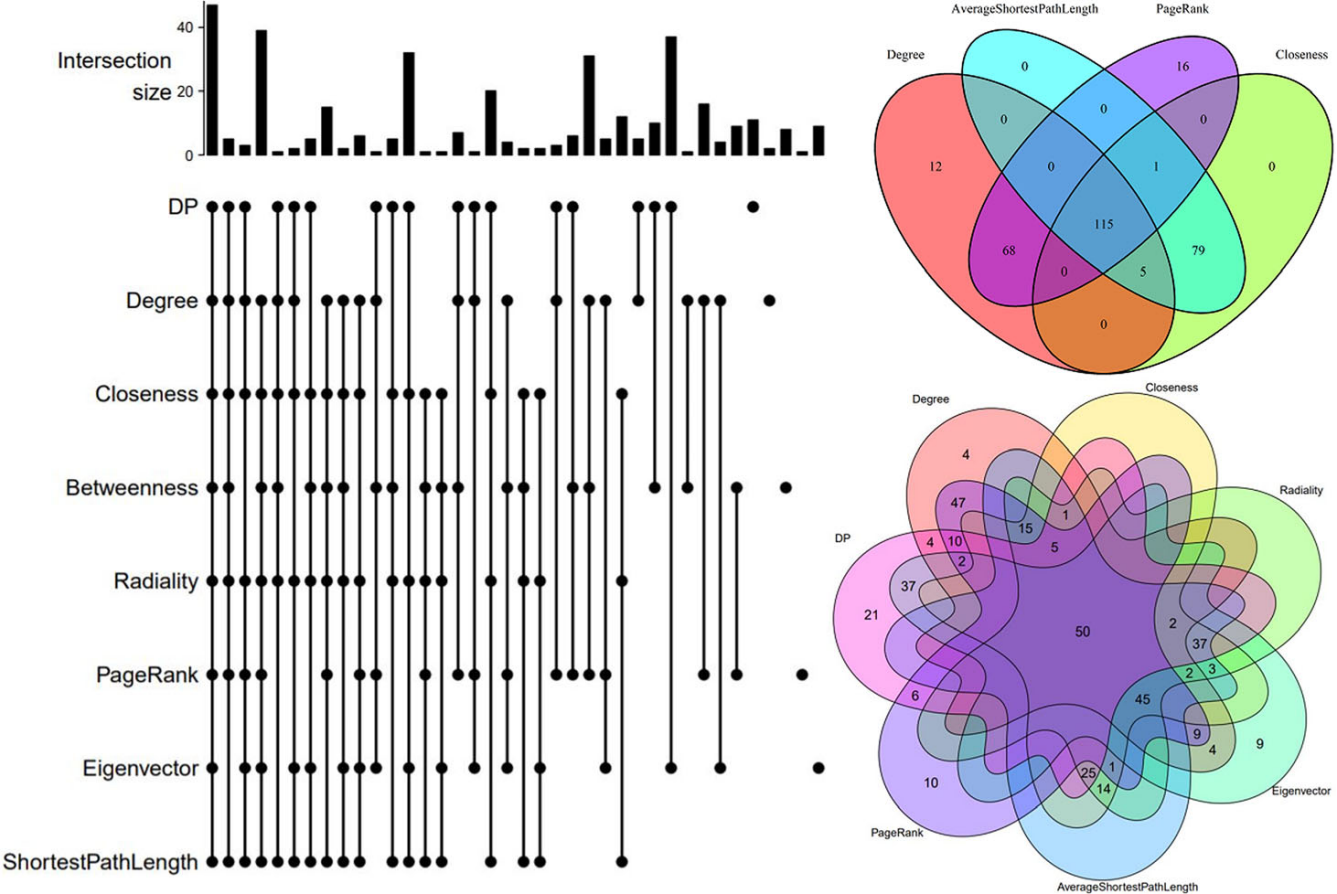
The pictures on the top and bottom right corner and the one on the left respectively show four, seven, and eight centralities with the most common central host proteins

Figure 14 summarizes the selections of the most central host proteins by different measures. For each of the 11 centrality measures worked in this article, 200 HPs with the highest centrality scores were selected. Degree, closeness, average shortest path, and page rank centralities had the most common HP targets among their first 200 highest score HPs.

By investigating all the centrality measures, we found that all of the top 10 HPs with the highest centrality scores in different centrality measures are essential proteins.

Moreover, the following HPs were detected as HPs with high centrality score in the most of the centrality measures:

- P05412 (Transcription factor AP-1)
- P62993 (Growth factor receptor-bound protein 2)
- P08238 (Heat shock protein HSP 90-beta)
- Q99459 (Cell division cycle 5-like protein)
- Q08379 (Golgin subfamily A member 2)
- P61981 (14–3-3 protein gamma)
- Q86VP6 (Cullin-associated NEDD8-dissociated protein 1)
- P10809 (60 kDa heat shock protein, mitochondrial)
- P11142 (Heat shock cognate 71 kDa protein)
- P27824 (Calnexin)

Connectivity of HPs targeted by the same VP and propagation speed of viruses in HPPIN reveals that VPs select their targets purposefully.

The tendency of virus proteins with high VC scores in targeting essential proteins and the results of DP centrality in detecting essential proteins shows that HPs with high DP score can be considered as potential drug targets.

Investigating Crown centrality scores reveals the tendency of VPs to have collaboration in targeting same HP pairs.

Finally, Table 7 summarizes some of the possible usages of the proposed centralities.

**Table 7:** Some of the usages of new proposed centralities

Centrality		
CC	Crown	Specifying the amount of cooperation between the two authors in writing articles, or the cooperation between viruses in targeting the same pair of HPs.
CI	Component Index	Detecting groups in social networks, or components of HPPIN.
PS	Propagation Speed	Revealing the speed of the rumor in society, or the speed of spreading virus in HPPIN.
DP	Diversity of Predator	Disclosing favorite HP targets of viruses.
VC	Vulnerable Centrality	Revealing the indispensable parts of a network, or the most important proteins of a virus which should be initially inhibited.
DSP	Decreased Shortest Path	Detecting critical points of a network, or potential drug targets.
CHTV	Connectivity of Human node Targeted by the same Virus node	Disclosing the clique like subnetworks, or the attack tendency of viruses.

## Conclusions

In this article, we studied the properties of a bipartite network generated from interactions between human proteins versus virus proteins. As there are different virus families, we investigated each family as a separate network and reported the results for most famous virus families. As HVPPIN is a bipartite network and centrality measures for this type of network is scarce, seven new centralities were proposed on HVPPIN and measured on different strains of famous virus families. In all proposed centralities, significant difference was observed between real and random samples’ scores.

Moreover, we found some significant properties of essential proteins. By investigating HPPIN and calculating ten famous centralities, it was revealed that essential HPs have a considerable higher centrality scores in comparison with non-essential HPs and it could be used for finding new essential HPs. In addition, we observed that DP scores have the same pattern. For finding the best marker of EPs, for each of the centralities, we select 200 HPs with the highest scores and calculated the sensitivity of detecting EPs with them. Results demonstrate that DP outperforms the others. Furthermore, analyzing VC scores of HVPPIN disclosed that VPs with high VC scores target essential HPs significantly higher than VCs with low VC scores. This observation could be used for choosing VCs with high VC score as the first target of drug design.

The current work can be extended by using the proposed centralities in the other bipartite networks. Moreover, it is suggested to recalculate centralities by adding new HPPIs to HPPIN for finding potential EPs. Doing the same with HVPPIN and considering new HP targets of high-VC-scores VPs and HPs with high DP scores as potential EPs are also recommended.

## Data Availability

Some part of data, analyzed during the current study are publicly available in the http://bioinf.modares.ac.ir/software/PHINA The other datasets which were analyzed during the current study available from the corresponding author on reasonable request.

## Abbreviations

HPPI: Human protein-protein interaction
HVPPI: Human-virus protein-protein interaction
HPPIN: Human protein-protein interaction network
HVPPIN: Human-virus protein-protein interaction network
HP: Human protein
VP: Virus protein
EP: Essential protein
CHTV: Connectivity of human proteins targeted by same virus protein
PS: Propagation speed
DP: Diversity of predators
DSP: Decreased shortest path
CI: Component index
CR: Crown centrality
VC: Vulnerable centrality
DC: Degree centrality
NC: Neighborhood connectivity
SP: Average shortest path length
TC: Topological coefficients
CS: Closeness centrality
CC: Clustering coefficients
BC: Betweenness centrality
RD: Radiality
EV: Eigenvector centrality
PR: Page rank centrality
HTV: Human proteins targeted by virus
ED: Evolutionary distance
ISG: Induced sub graph
DEG: Differentially expressed gene
